# Behçet’s disease unraveled: Insights into clinical manifestations, diagnosis, and management

**DOI:** 10.1097/MD.0000000000044614

**Published:** 2026-05-12

**Authors:** Mansour Alghamdi, Stephen Lindsey

**Affiliations:** a Department of Medicine, Louisiana State University Health Science Center, New Orleans, LA

**Keywords:** Behçet’s disease, clinical manifestations, diagnostic criteria, epidemiology, management, pathogenesis

## Abstract

Behçet’s disease is a rare and recurrent multisystem inflammatory disorder, primarily distinguished by systemic vasculitis that affects veins and arteries of various sizes. Clinically, Behçet’s disease is characterized by the presence of recurrent oral and genital ulcers, skin lesions, and uveitis. The prevalence of this condition is significantly elevated along the historical Silk Road, particularly in Turkey; however, epidemiological data indicate variations across different regions and ethnic groups. The diagnosis of Behçet’s disease is typically established during the third decade of life, with a more severe prognosis noted when the onset occurs before the age of 25. This review aims to explore the epidemiology, pathogenesis, clinical manifestations, diagnostic criteria, and current therapeutic strategies relevant to Behçet’s disease. Specific attention is devoted to understanding the global distribution of the disease, its associated morbidity patterns, and current management and treatment modalities.

## 1. Introduction

Behçet’s disease is a recurrent, multisystem inflammatory disorder that affects various organs, including the skin, mucous membranes, eyes, joints, gastrointestinal tract, and central nervous system. It is primarily characterized by systemic vasculitis, with a predominant involvement of veins rather than arteries. Also referred to as Behçet’s disease, this rare condition triggers widespread inflammation in blood vessels throughout the body and is clinically identified by recurrent oral and genital ulcers, skin lesions, and uveitis.^[1]^

Although Behçet’s disease is observed worldwide, it has a higher prevalence in regions along the historic Silk Road, spanning from East Asia to the Mediterranean Basin.^[2]^ In recent decades, significant advancements have improved our understanding of Behçet’s disease, which is now recognized as a primary systemic vasculitis that can affect blood vessels of all sizes, including both venous and arterial systems.^[3]^

The epidemiology of Behçet’s disease varies considerably depending on ethnicity and geographic location. Turkey reports the highest prevalence, with 420 cases per 100,000 individuals. In Europe, a distinct north-south gradient exists, with prevalence rates ranging from 0.3 to 4.9 cases per 100,000 in northern countries and from 1.5 to 15.9 cases per 100,000 in southern regions. In the United States, the prevalence is estimated at 5.2 cases per 100,000 individuals.^[4]^

This review will examine the epidemiology and pathogenesis of Behçet’s disease, with a focus on its clinical manifestations, diagnostic criteria, and current therapeutic approaches.

## 2. Epidemiology

Behçet’s disease typically manifests in the third decade of life and is rarely seen before puberty or in older adults. Early onset, defined as presentation before age 25, is associated with a poorer prognosis. Although Behçet’s disease affects both sexes equally, the male-to-female ratio varies based on dominant clinical features and ethnic background.^[5]^ Males generally experience a more severe disease course, with a higher incidence of ocular, vascular, and neurologic complications. In contrast, females are more commonly affected in non-endemic regions, where the disease often presents in a milder form, suggesting that environmental factors may influence disease severity.^[6]^

A recent epidemiological study of Behçet’s disease in the United States, involving 1323 patients, represents the most significant U.S. cohort of Behçet’s disease to date. The findings confirmed a higher prevalence of the disease among females, consistent with previous reports, and revealed a significant increase in the use of Tumor Necrosis Factor-Alpha (TNF-alpha) inhibitors as biological therapies, exceeding previous records. U.S. patients were generally older and had a higher utilization of biologic medications compared to those in endemic regions, suggesting potential epidemiological and clinical differences. In contrast, studies from Asia and the Middle East reported a predominance of male patients and a lower use of biologic therapies.^[7,8]^

A recent systematic literature review, which included data from 45 population-based surveys, provided the most accurate estimates of the global prevalence of Behçet’s disease. The overall global prevalence was estimated at 10.3 cases per 100,000 individuals. Region-specific estimates were 119.8 per 100,000 (95% CI: 59.8–239.9) for Turkey, 31.8 per 100,000 (95% CI: 12.9–78.4) for the Middle East, 4.5 per 100,000 (95% CI: 2.2–9.4) for Asia, 5.3 per 100,000 (95% CI: 3.4–8.2) for Southern Europe, 2.1 per 100,000 (95% CI: 1.1–4.0) for Northern Europe, and 3.8 per 100,000 (95% CI: 2.2–6.8) for North America and the Caribbean Islands.^[9,10]^

## 3. Pathogenesis

Recent research indicates that Behçet’s disease may be classified as a complex autoinflammatory syndrome. The most significant genetic risk factor identified is the HLA-B*51/B5 allele, although other HLA-independent loci also contribute to disease susceptibility. Key pathogenic elements include hyper-reactivity to *Streptococcus sanguinis* antigens and changes in the microbiome. Neutrophil hyperactivity, characterized by increased oxidative burst, chemotaxis, and the formation of neutrophil extracellular traps, is a defining characteristic of Behçet’s disease.^[11]^

A large meta-analysis revealed that carriers of the HLA–B51/B5 allele have a significantly heightened risk of developing Behçet’s disease, with an odds ratio of 5.78 compared to noncarriers. This association has been consistently observed across various geographic regions and ethnic groups, indicating that HLA-B51/B5 is a primary and causal risk factor for the condition. The meta-analysis, which included 4800 patients with Behçet’s disease and 16,289 controls, demonstrated moderate heterogeneity, likely due to the differing proportions of male patients with Behçet’s disease. The population-attributable risk for Behçet’s disease associated with HLA–B51/B5 ranged from 32% to 52% across various regions. Additionally, variations in risk based on sex suggest potential interactions between the HLA–B51/B5 allele and specific characteristics of Behçet’s disease, further underscoring its role as a major genetic determinant in the pathogenesis of Behçet’s disease.^[12]^

The innate immune system plays a crucial role in Behçet’s disease, particularly involving Toll-like receptors in monocytes. Hyperactive neutrophils exhibit increased chemotaxis, the production of reactive oxygen species, and the formation of neutrophil extracellular traps. Elevated pro-inflammatory cytokines such as IL-1β, TNF-α, and IL-6 reflect this heightened innate immune activation. In particular, activation of the NLRP3 inflammasome contributes to the excessive release of IL-1β.^[13]^

**Figure 1.**
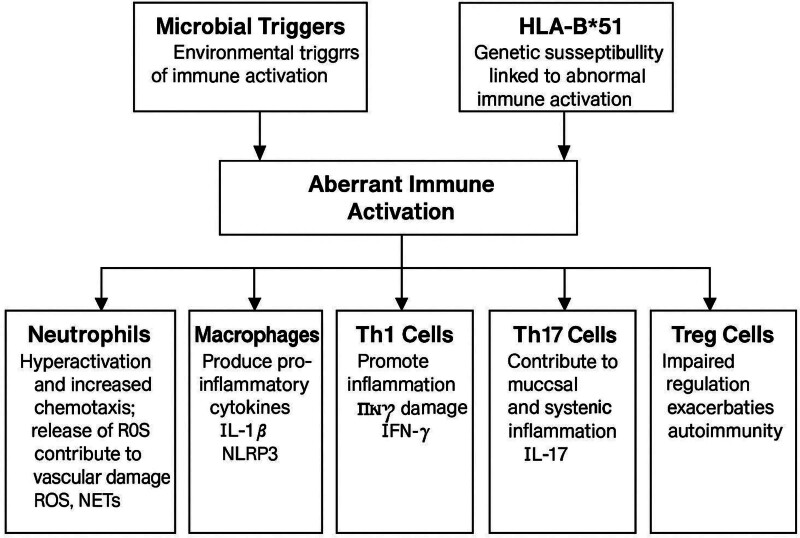
Summarizing the immunopathogenesis of Behçet’s disease. HLA-B*51 = human leukocyte antigen B*51, IFN-γ = interferon-gamma, IL-1β = interleukin-1β, IL-17 = interleukin-17, NLRP3 = NOD-, LRR-, and pyrin domain-containing protein 3 inflammasome, ROS = reactive oxygen species.

The adaptive immune system is also significantly involved, characterized by an imbalance in T helper cell subsets. Th1 cells secrete IFN-γ and TNF-α, promoting chronic inflammation, while Th17 cells produce IL-17, which further recruits neutrophils and amplifies the inflammatory response. Regulatory T-cells (Tregs), which normally suppress excessive immune activity, are often functionally impaired in Behçet’s disease, failing to control these inflammatory pathways.^[14]^ See Figure 1.

The classification of Behçet’s disease illustrates its complex pathogenesis, displaying features of autoinflammatory, autoimmune, and spondyloarthropathy-like disorders. Similar to autoimmune diseases, Behçet’s disease involves T-cell and B-cell responses to microbial antigens and responds to immunosuppressive therapies such as azathioprine and ciclosporin. However, in contrast to classic autoimmune diseases, Behçet’s disease does not exhibit a female predominance or the presence of autoantibodies. Its recurrent mucocutaneous lesions and nondeforming arthritis associate it with autoinflammatory disorders, driven by neutrophil-mediated inflammation, elevated proinflammatory cytokines, and genetic variants in the *MEFV* gene. Nonetheless, Behçet’s disease typically appears after puberty and shows a limited response to anti-IL-1 therapies.^[15]^

## 4. Diagnostic criteria

Diagnosing Behçet’s disease remains challenging due to the lack of specific biological or histological markers, with the diagnosis primarily relying on clinical presentation and imaging. The most used classification systems are the International Study Group (ISG) 1990 criteria^[16]^ and the International Criteria for Behçet’s Disease (ICBD) 2014.^[17]^ Both highlight recurrent oral ulceration as a key feature, along with other clinical manifestations such as genital ulceration, ocular and skin lesions, and a positive pathergy test (Table 1). Important diagnostic indicators include genital scarring, characteristic eye involvement, major vascular involvement, and neurological lesions. Although HLA-B51 is prevalent in certain ethnic groups, it is not a definitive diagnostic marker for the condition. Differential diagnoses must consider conditions such as nutritional deficiencies, infections, autoimmune disorders, and inflammatory bowel diseases. Severe manifestations may occur in isolation, meaning that fulfilling all criteria should not be mandatory to initiate treatment. Therefore, diagnosing Behçet’s disease necessitates a careful assessment of symptoms, exclusion of alternative conditions, and the use of classification criteria as a structured but cautious guide in clinical practice.^[4]^

**Table 1:** Behçet’s disease diagnostic criteria.

Criteria	Requirements	Sensitivity	Specificity
International Study Group criteria, 1990^[14]^	Recurrent major, minor aphthous, or herpetiform oral ulceration plus 2 manifestations of recurrent genital, ocular, or skin lesions, or positive pathergy test.	90.0% (95% CI 88.8–91.2)16	98.8% (95% CI 98.3–99.3)16
International Criteria for Behçet’s Disease, 2014^[15]^	Two points for either oral ulcers, genital ulcers, or ocular lesions. One point for cutaneous, neurological, or vascular involvement, or a positive pathergy test. Score of at least 4 points needed for Behçet’s disease diagnosis	97.6% (95% CI 96.9–98.2)16	90.8% (95% CI 89.4–92.1)16

A recent study evaluating the sensitivity and specificity of diagnostic criteria for Behçet’s disease found that while the ISG criteria exhibit higher specificity, they are less sensitive compared to the ICBD criteria. Modifying the ICBD threshold to 5 points enhanced both sensitivity and specificity, achieving nearly perfect concordance with the ISG criteria. This adjustment lowers the likelihood of false positives in the ICBD, which is influenced by the high prevalence of oral aphthosis. A 5-point threshold may improve diagnostic accuracy, particularly in patients with uveitis, while addressing limitations of the ISG criteria, such as lower sensitivity and the exclusion of vascular and neurological manifestations.^[18]^

## 5. Clinical features

Mucocutaneous manifestations are the primary clinical indicators of Behçet’s Disease. The most common symptoms include oral ulcers, which occur in 92% to 100% of cases, followed by genital ulcers in 57% to 93%, and cutaneous vasculitic lesions in 38% to 99% of patients. Ocular involvement is noted in 29% to 100% of cases, while articular manifestations occur in 16% to 84% of cases. Oral ulcers often present as the initial clinical manifestation, though genital ulcers and EN-like lesions may also indicate the onset of the disease. In some patients, the clinical presentation may be limited to mucocutaneous symptoms, with systemic organ involvement potentially developing months or years later.^[19]^

### 5.1. Oral ulcers

**Figure 2.**
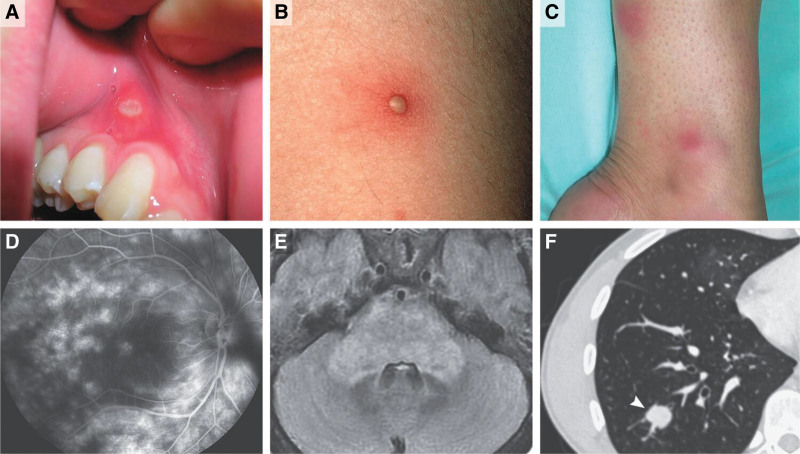
Clinical Manifestations of Behçet’s Syndrome. (A) oral ulcer. (B) a papulopustular lesion. (C) erythema nodosum (EN). (D) capillary impairment and vascular leakage on retinal fluorescein angiography. (E) a brain-stem inflammatory lesion on a T2-weighted axial magnetic resonance imaging sequence, with an edematous, protuberant FLAIR (fluid-attenuated inversion recovery) hypersignal extending to the middle cerebellar peduncles and discrete hypertrophy. (F) aneurysmal dilatation of a pulmonary-artery branch (arrowhead). Saadoun D, et al. Behçet’s Syndrome. N Engl J Med. 2024 Feb 15;390(7):640–651. doi: 10.1056/NEJMra2305712. PMID: 38354143.*.*With permission from the New England Journal of Medicine (NEJM), License Number 6075080861718.

Oral ulcers associated with Behçet’s disease are painful aphthous lesions that can significantly impact eating, drinking, speaking, and swallowing. These ulcers initially present as erythematous, vesiculopustular lesions and evolve into oval or round ulcers within 24 to 48 hours. They typically develop on nonkeratinizing mucosal surfaces, including the lips, buccal mucosa, ventral and lateral surfaces of the tongue, the floor of the mouth, and the soft palate (Fig. 2).

Oral ulcers are categorized into 3 types based on size and number:

1. **Minor**: The most prevalent type (80% to 85%), characterized by small (<1 cm), shallow ulcers that typically heal within 1 to 2 weeks without leaving scars.
2. **Major**: Less common (10% to 15%), larger (>1 cm), deeper, and more painful ulcers that frequently heal with scarring and tissue loss, potentially taking up to 6 weeks to resolve.
3. **Herpetiform**: The rarest form (5%), consisting of numerous small ulcers (1–3 mm in size, 10–100 total).

Oral ulcers generally heal spontaneously within 1 to 3 weeks, though major oral ulcers may take longer. They recur unpredictably in days to months, and local traumas can trigger recurrences through a mucosal pathergy reaction.^[19]^

### 5.2. Genital ulcers

Genital ulcers represent a distinctive and specific feature of Behçet’s disease and may occasionally serve as the initial manifestation. Although their appearance and progression resemble those of oral ulcers, genital ulcers are typically deeper and recur less frequently. Due to their depth, these ulcers often heal with scarring, making it essential to assess for scars from previous lesions in patients suspected of having Behçet’s disease. In males, approximately 90% of genital ulcers occur on the scrotum, while lesions are rarely found on the glans or corpus of the penis. In females, genital ulcers primarily localize to the labia, though they may also appear in the vulva, vagina, and cervix. Early recognition of genital ulcers can enable timely diagnosis and management of Behçet’s disease.^[20]^

Throughout the extended course of Behçet’s disease, testicular and epididymal involvement, marked by scrotal pain and swelling, has been reported. The incidence of epididymo-orchitis varies by age, with a prevalence of 25% in juveniles and 8.9% in adults. Patients with this condition often show higher rates of cutaneous involvement, genital ulcers, arthritis, central nervous system manifestations, and positive pathergy tests compared to individuals without epididymo-orchitis. Typically, epididymo-orchitis develops several years after the onset of Behçet’s disease, often accompanied by a range of clinical symptoms.^[21]^ A study shows that patients with epididymitis frequently experience more severe manifestations of Behçet’s disease, highlighting the need for comprehensive evaluation and management by healthcare providers.^[22]^

### 5.3. Cutaneous manifestations

Cutaneous findings in Behçet’s disease are diverse, with common lesions including papulopustular eruptions, EN-like nodules, and superficial thrombophlebitis. Pathergy testing can aid diagnosis, and rare manifestations also occur, mimicking other neutrophilic dermatoses.

#### 5.3.1. Papulopustular lesions

The most common cutaneous manifestation of Behçet’s disease, identified in 65% to 96% of cases, is folliculitis, characterized by sterile, acne-like pustules situated on an erythematous base. These pustules typically originate as papules and progress to pustules within 24 to 48 hours. They commonly appear on the trunk, lower extremities, and face (Fig. 2). It is essential to differentiate these pustules from those seen in acne vulgaris or folliculitis.^[20]^

Biopsies from non-follicular pustules found on non-seborrheic areas, such as the trunk or extremities, show greater specificity and often reveal immune complex-mediated vasculitis, a characteristic feature of cutaneous lesions in Behçet’s disease.^[23]^ Boyvat et al documented neutrophilic vasculitis in non-follicular pustules, reinforcing their specificity to Behçet’s disease.^[24]^ Furthermore, these pustules seem to be more common in patients with Behçet’s disease who also have arthritis, indicating a potential connection between Behçet’s disease-related arthritis and acne-associated arthritis.^[25]^

#### 5.3.2. Erythema Nodosum-like lesions

Approximately one to two third of patients with Behçet’s disease (15% to 78%) develop erythema nodosum (EN)-like lesions, which are more prevalent in females (Fig. 2). These lesions are identified by painful, red, subcutaneous nodules that typically appear on the lower extremities, especially the legs. EN-like lesions do not ulcerate and typically resolve within 2 to 4 weeks, often leaving post-inflammatory hyperpigmentation in individuals with pigmented skin; however, recurrences are common.^[19]^

Histopathologically, EN-like lesions in Behçet’s disease are characterized by panniculitis, typically exhibiting lobular or mixed septal and lobular patterns. These lesions contain varying quantities of neutrophils, lymphocytes, histiocytes, and necrotic adipocytes. Unlike classic EN, vasculitis is often seen in EN-like lesions associated with Behçet’s disease, with evidence of lymphocytic vasculitis and areas of leukocytoclastic vasculitis, which may be accompanied by phlebitis or arteriolitis.^[26]^

Furthermore, EN-like skin lesions in Behçet’s disease show a marked delayed hypersensitivity reaction. The inflammatory infiltrate primarily comprises activated T-cells and macrophages, along with natural killer cells. These observations suggest that delayed hypersensitivity reactions, involving antigen-antibody-mediated cytotoxicity, may significantly contribute to the pathogenesis of Behçet’s disease lesions.^[27]^

The presence of EN-like lesions may indicate a milder form of Behçet’s disease; however, significant vasculitis, particularly phlebitis, within these lesions may suggest gastrointestinal involvement. A study of 78 patients with EN-like lesions and superficial thrombophlebitis indicated that these skin manifestations could precede serious visceral complications.^[28,29]^

#### 5.3.3. Superficial thrombophlebitis

Superficial thrombophlebitis is the most common vascular lesion associated with Behçet’s disease, presenting as a painful, erythematous, cord-like lesion. When thrombosis occurs in smaller veins, it can be challenging to visually distinguish it from lesions resembling EN. Ultrasonography is a valuable tool for this differentiation, while biopsy can provide a definitive diagnosis.

Accurately distinguishing between these lesions is crucial due to the established relationship between superficial thrombophlebitis and deep vein thrombosis (DVT). Similar to EN-like skin lesions, superficial thrombophlebitis may precede serious visceral complications^[28,29]^ and is considered a warning sign for the potential development of more severe venous thrombosis.^[1]^

### 5.4. Pathergy

Skin pathergy is a hyperreactive response to needle-induced trauma, characterized by the formation of a papule or pustule 24 to 48 hours after a sterile needle prick. This response involves the activation of both the innate and adaptive immune systems, with an inflammatory reaction mediated by polymorphonuclear cells and T-cells.^[30]^

Several factors, including male gender, active disease state, and the presence of oral aphthosis, pseudofolliculitis, and uveitis, influence Pathergy reactivity. Increased reactivity is also observed in males and specific patient populations. There is considerable variation in pathergy reactivity across different demographics, with lower positivity rates reported in Northern Europe and higher rates among communities along the historical Silk Road, such as Turkey, China, and various Middle Eastern countries.^[30]^

The test procedure involves using sharp or blunted 20-gauge needles to puncture the cleansed forearms (typically prepped with alcohol) perpendicularly or obliquely. To enhance trauma, needles may be twisted during the procedure. Physicians generally perform 2 to 3 punctures on each forearm.^[30]^

Although the sensitivity of the skin pathergy test has declined over the years, its specificity remains relatively high. Reactivity is not consistent throughout the disease course and can be positive in other conditions such as Sweet syndrome, pyoderma gangrenosum, Crohn disease, A20 haploinsufficiency, and interleukin-1 receptor antagonist deficiency.^[31]^

Notably, the combination of a 23-valent polysaccharide pneumococcal vaccine with a 20-gauge needle prick has shown improved sensitivity (64.3%) and specificity (100%) compared to standard testing methods, especially in patients with active Behçet’s disease. This innovative approach highlights the potential of the 23-valent polysaccharide pneumococcal vaccine as a more effective diagnostic tool for Behçet’s disease due to its enhanced sensitivity relative to traditional methods.^[32]^

### 5.5. Other cutaneous manifestations

Less common cutaneous manifestations of Behçet’s disease include skin ulcers, Sweet syndrome-like lesions, pyoderma gangrenosum-like lesions, erythema multiforme-like lesions, pernio-like lesions, palpable purpura, Henoch-Schönlein purpura, bullous necrotizing vasculitis, subungual infarctions, hemorrhagic bullae, furuncles, abscesses, and acral purpuric papulonodular lesions. Histologically, these lesions are marked by neutrophilic infiltrates, classifying them as neutrophilic dermatoses. Skin ulcers in Behçet’s disease are typically deep, punched-out lesions with red and edematous margins and a yellow necrotic base. These ulcers are commonly located in the armpits, thighs, breasts, neck, groin area, and between the toes. They tend to recur and often leave scars. Additionally, leg ulcers in Behçet’s disease may be associated with DVT.^[19]^

Sweet syndrome-like lesions appear as painful erythematous nodules or plaques on the face, neck, and extremities, often accompanied by fever and leukocytosis. The clinical and histological similarities between Sweet syndrome and Behçet’s disease require careful differentiation to ensure accurate diagnosis and appropriate treatment.^[20]^

### 5.6. Articular involvement

Joint involvement affects approximately 50% of patients with Behçet’s disease who present with arthritis or arthralgia. It typically manifests as mono- or oligoarticular and occasionally symmetrical. This joint disease usually resolves within weeks and rarely results in deformities or radiological erosions; however, chronic arthritis and osteonecrosis may occur in some cases.^[33]^

The most affected joints are the knees, ankles, wrists, and elbows. Synovial analysis reveals neutrophilic and mononuclear inflammatory infiltrates, often associated with thrombosis of small vessels. Ankylosing spondylitis has been reported in 10% of cases, with pseudofolliculitis identified as the most frequently associated manifestation.^[34]^ In a study of 37 patients with Behçet’s disease in Turkey, the radiographic incidence of sacroiliitis did not exceed that of 28 age- and sex-matched controls.^[35]^

Furthermore, a study involving 44 patients with Behçet’s disease and arthritis indicated that acneiform skin lesions, such as papules and pustules, were observed more frequently in this cohort. This suggests a potential association between arthritis in Behçet’s disease and acne-associated arthritis.^[25]^ Additionally, patients exhibiting both arthritis and acne lesions demonstrate significantly higher enthesopathy scores compared to those without acne, underscoring a possible connection between these conditions.^[36]^

### 5.7. Ocular involvement

The diagnosis of Behçet’s disease often relies on the presence of ocular complications, which can be severe and may result in vision loss. The disease is characterized by recurrent episodes of ocular inflammation, often presenting as anterior uveitis or retinal vasculitis.^[37]^

#### 5.7.1. Uveitis and retinal vasculitis:

Behçet’s uveitis typically arises in the late 20s and is more common in males. The condition tends to be more severe in men, which correlates with a higher risk of vision loss. In a study involving 880 patients, ocular involvement was bilateral in 78.1% of cases and unilateral in 21.9% of cases. Panuveitis was identified as the most prevalent type of uveitis in both sexes. Although isolated anterior uveitis is rare, it often initially presents as unilateral, but can subsequently affect both eyes. Hypopyon, once considered a hallmark of Behçet’s disease, is now observed less frequently than fundus lesions. A characteristic pattern of sudden onset followed by improvement and subsequent recurrence of inflammation is a strong indicator of Behçet’s uveitis, helping to distinguish it from other conditions^[38]^ (Fig. 2).

Ocular involvement in Behçet’s disease, primarily manifesting as uveitis, is characterized as an independent cluster with a low likelihood of association with other systemic involvements, such as gastrointestinal, cardiovascular, or central nervous system manifestations. Nongranulomatous anterior uveitis is the most common ocular manifestation of Behçet’s disease. Additionally, concomitant cutaneous manifestations, including papulopustular lesions and EN, are often seen in patients with Behçet’s uveitis, affecting approximately 40% to 60% of individuals.^[38]^

Posterior or panuveitis is the most common form of posterior segment involvement, often associated with retinal vasculitis and vitritis. Macular edema is the most common complication associated with this condition. Fundus lesions and sight-threatening complications occur more frequently in males. Complete resolution between inflammatory episodes is uncommon, and the severity and frequency of posterior segment inflammatory attacks significantly affect the extent of permanent structural changes and the rate of irreversible vision loss.^[39,40]^

### 5.8. Vascular involvement

Behçet’s disease frequently involves the vascular system, resulting in various thrombotic and aneurysmal conditions. The most frequent vascular complications include superficial and DVT, impacting up to 50% of patients. However, Behçet’s disease is also marked by venous thrombosis in unusual locations, such as the inferior and superior vena cava, suprahepatic veins (Budd-Chiari syndrome), portal vein, cerebral sinuses, and even the right atrium and ventricle. Arterial involvement, including in situ thrombosis and aneurysms affecting the pulmonary arteries, abdominal aorta, and peripheral and visceral arteries, is another distinctive feature of this disease.^[41]^

A study involving 796 patients with Behçet’s disease found that vascular involvement was present in 12.8% of cases. Males were more frequently affected than females, with a male-to-female ratio of 3.86:1. The average age at which vascular involvement began was 29.5 years. Notably, a vascular lesion was the first clinical manifestation of Behçet’s disease in 27.5% of patients. Women with Behçet’s disease were more likely to develop arterial lesions, while men were more predisposed to venous lesions. Furthermore, patients with vascular involvement exhibited significantly lower rates of ocular involvement, genital ulcers, and arthritis compared to those without vascular complications.^[42]^

In another study involving 883 patients, individuals with extrapulmonary artery involvement were notably older than those with venous or pulmonary-artery issues. The study identified significant correlations among various conditions: dural sinus thrombosis was associated with pulmonary-artery involvement, Budd-Chiari syndrome was linked to both inferior vena cava syndrome and superior vena cava syndrome, and a connection was established between inferior vena cava syndrome and superior vena cava syndrome.^[43]^ Survival rates were primarily influenced by the extent of thrombosis in the inferior vena cava rather than merely the presence of thrombosis in the hepatic veins.^[44]^

#### 5.8.1. Pulmonary-artery aneurysms and Hughes-Stovin syndrome

Pulmonary-artery aneurysms (PAAs) are the most common pulmonary lesions associated with Behçet’s disease and are often associated with hemoptysis (Fig. 2). The underlying pathophysiology includes pulmonary vasculitis, which can lead to thrombosis, infarction, hemorrhage, and the formation of PAAs. Patients with subtle or unclear radiologic findings should be closely monitored, as it is crucial to distinguish Behçet’s disease-related pulmonary complications from pulmonary thromboembolic disease. Reports have indicated fatalities in Behçet’s disease patients shortly after the initiation of anticoagulant or thrombolytic therapies.^[45]^

PAAs are more frequently observed in males and are typically diagnosed around age 29. The most common symptom is hemoptysis, followed by cough, fever, dyspnea, and chest pain. In a study of 47 patients, over 90% showed active disease in other organ systems at the time of PAA diagnosis. Most patients presented with lung nodules or cavities. Peripheral venous thrombosis was often noted, with some patients also exhibiting intracardiac thrombi. PAAs frequently affect multiple sites, primarily involving the descending branches of the pulmonary artery.^[46]^

Hughes-Stovin Syndrome (HSS) is a very rare clinical disorder regarded as a variant of Behçet’s disease. It is characterized by thrombophlebitis and the presence of multiple pulmonary and/or bronchial aneurysms. Typical symptoms consist of cough, dyspnea, fever, chest pain, and hemoptysis. The exact etiology and pathogenesis of HSS remain unclear; however, potential contributing factors may include infections and angiodysplasia. The prognosis for HSS is generally poor, with aneurysmal rupture identified as the leading cause of mortality.^[47]^

### 5.9. Neurological involvement

Neuro-Behçet’s disease (NBD) can be categorized into 2 main subtypes: parenchymal and non-parenchymal.

**Parenchymal NBD** is characterized by an inflammatory meningoencephalitis process. It typically presents with a sub-acute onset of brain-stem syndrome, which may be accompanied by additional features such as cerebral hemispheric or spinal cord syndromes. Common symptoms include pyramidal weakness, behavioral changes, headaches, ophthalmoplegia, and sphincter dysfunction. The disease course can be either relapsing-remitting or progressively worsening, categorized as primary or secondary progressive (Fig. 2).

**Non-parenchymal NBD** is primarily associated with vascular involvement. It often manifests as headaches and visual disturbances resulting from intracranial hypertension, usually resulting from cerebral venous thrombosis. Less frequently, it may present as an acute stroke caused by arterial thrombosis, dissection, or aneurysm.^[48,49]^

Spinal cord involvement is a rare complication of Behçet’s disease. When it occurs, it is typically characterized by longitudinally extensive transverse myelitis. A retrospective review of 10 cases of spinal cord involvement across 7 patients with Behçet’s disease identified longitudinally extensive transverse myelitis as a key feature. This condition usually affects the cervical spinal cord, with inflammatory lesions resembling myelitis extending over 2 or more segments and potentially continuing into the brainstem.^[50,51]^ Notably, the incidence of new-onset neuro-Behçet’s disease was significantly higher in patients treated with cyclosporine A compared to those receiving other therapeutic agents.^[52]^

### 5.10. Gastrointestinal involvement

Gastrointestinal manifestations of Behçet’s disease are particularly noteworthy because of their association with significant morbidity and mortality. Common symptoms include abdominal pain, nausea, vomiting, diarrhea, and gastrointestinal bleeding. The hallmark of gastrointestinal involvement is intestinal ulceration, which is believed to result from small-vessel vasculitis. Behçet’s disease can affect any segment of the gastrointestinal tract, with the terminal ileum being the most commonly affected area, followed by the ileocecal valve, cecum, and colon.^[53,54]^

## 6. Management

The primary objective of treating Behçet’s disease is to achieve and sustain remission while enhancing the patient’s quality of life. Treatment strategies are typically tailored to the specific symptoms and severity of the condition (Table 2). Due to the limited availability of high-quality clinical trials, treatment recommendations are often based on case reports, case series, and a few randomized controlled trials. Consequently, therapeutic approaches should be individualized, taking into account age, gender, the type and severity of organ involvement, and patient preferences.^[55]^

**Table 2:** Management.

Clinical condition	Therapy
Mucocutaneous involvement	Corticosteroids; topical/oral therapies
	Antiseptics/antibiotics
	Sucralfate
	Pentoxifylline
	Colchicine
	Apremilast
	Azathioprine
	Thalidomide
	TNF-alpha inhibitors
	Interferon-alpha
Eye involvement	Corticosteroids with systemic therapy
	Azathioprine
	Cyclosporine A
	Interferon-alpha
	TNF-alpha inhibitors
Vascular involvement including venous thrombosis	Corticosteroids
	Azathioprine
	Cyclophosphamide
	Cyclosporine A
	TNF-alpha inhibitors
	Anticoagulants is permissible if there is a low risk of bleeding and pulmonary-artery aneurysms are excluded.
Neurological involvement	Corticosteroids
	Azathioprine
	TNF-alpha inhibitors Cyclophosphamide.
	Mycophenolate mofetil, methotrexate and tocilizumab may be considered.
	Cyclosporine A should be avoided
Articular involvement	Corticosteroids
	Colchicine
	Azathioprine
	TNF-alpha inhibitors
Gastrointestinal involvement	Corticosteroids
	5-ASA
	Azathioprine
	TNF-alpha inhibitors
	Thalidomide

5-ASA = 5-aminosalicylic acid, TNF-alpha = tumor necrosis factor-alpha.

### 6.1. Mucocutaneous involvement

Topical therapies play a crucial role in managing Behçet’s disease, particularly regarding oral and genital ulcers. Corticosteroids, such as triamcinolone acetonide in oral paste or dexamethasone ointment, are effective for treating oral ulcers. For genital ulcers, combining corticosteroids with antiseptics such as fusidic acid and betamethasone is particularly beneficial, especially when applied in the early stages of lesion development. Sucralfate can help reduce pain and promote healing for both oral and genital ulcers. At the same time, pentoxifylline 5% gel has demonstrated efficacy in shortening the duration and alleviating pain associated with oral ulcers. Given that oral ulcers in Behçet’s disease resemble recurrent aphthous stomatitis, treatments commonly used for aphthous ulcers, including topical antibiotics (e.g., tetracyclines), antimicrobial agents (like chlorhexidine), amlexanox, and triclosan, can also be applied to Behçet’s disease lesions to facilitate healing.^[56]^

Colchicine has been a key treatment for Behçet’s disease since the 1980s, supported by several placebo-controlled trials that have explored its effects on mucocutaneous symptoms. Given its safety and favorable tolerability profile, colchicine is recommended as a first-line option for patients with isolated mucocutaneous involvement. While its effectiveness for oral ulcers and papulopustular lesions remains debated, colchicine has demonstrated efficacy for genital ulcers and nodular lesions, particularly in female patients.^[57]^

For patients with Behçet’s disease who continue to experience lesions despite colchicine therapy, alternative treatment options include immunomodulatory and immunosuppressive agents like azathioprine, thalidomide, and interferon-alpha. TNF-alpha inhibitors and apremilast may also be considered.^[58]^ Apremilast has been demonstrated to effectively reduce the frequency of oral ulcers compared to placebo, although it may induce side effects such as diarrhea, nausea, and headache.^[59]^

Additionally, dapsone and azithromycin have shown positive results in treating Behçet’s disease. Among the newer biological agents, interleukin (IL)-1 inhibitors, such as anakinra and canakinumab, have demonstrated partial benefits for mucocutaneous symptoms. However, IL-17 inhibitors such as secukinumab have proven ineffective, while IL-6 inhibitors, including tocilizumab, have been associated with the exacerbation of mucocutaneous lesions.^[58]^

### 6.2. Eye involvement

Isolated anterior uveitis in Behçet’s disease can be managed effectively with topical treatments. However, for patients exhibiting poor prognostic factors – such as a younger age, male sex, and early disease onset – systemic immunosuppressive therapy should be considered. Posterior uveitis requires a more aggressive treatment approach, typically involving conventional immunosuppressants or biological agents. Patients with posterior segment involvement should receive a regimen that includes azathioprine, cyclosporine A, interferon-alpha, or monoclonal anti-TNF antibodies. Systemic glucocorticoids should be administered only in conjunction with azathioprine or other systemic immunosuppressants.^[58]^

Clinical trials have shown that azathioprine and cyclosporine A effectively preserve vision and prevent flare-ups associated with uveitis in Behçet’s syndrome. However, reaching a consensus on managing patients who are refractory to these agents presents a significant challenge. In such cases, biological therapies, including TNF-alpha inhibitors and interferon-alpha, may be considered, taking into account factors such as infection risk, tolerability, and reimbursement considerations. Infliximab is frequently recommended for acute, sight-threatening situations, while adalimumab is preferred for long-term maintenance therapy. For patients experiencing unilateral or asymmetrical relapses, particularly those with cystoid macular edema, local therapy is advisable after stabilization of systemic treatment. This may include intravitreal injections of a dexamethasone implant, which has been shown to provide efficacy for a duration of 3 to 6 months.^[1]^

### 6.3. Vascular involvement

The management of DVT in Behçet’s disease primarily emphasizes the use of immunosuppressants, although the role of anticoagulants remains debated.^[4]^ According to EULAR guidelines, acute DVT in Behçet’s disease should be treated with glucocorticoids and immunosuppressants such as azathioprine, cyclophosphamide, or cyclosporine A. In refractory cases, monoclonal anti-TNF antibodies may be an option. The addition of anticoagulants is acceptable if there is a low risk of bleeding and PAAs are excluded.^[56]^ Immunosuppressive agents have demonstrated high efficacy in reducing the recurrence of venous thrombosis in this condition.^[60]^

For pulmonary-artery involvement, the recommended initial treatment is monthly pulse cyclophosphamide combined with corticosteroids. Should this approach prove ineffective, anti-TNF agents may be considered.^[61]^ An observational cohort study assessing the safety and efficacy of the IL-6 receptor inhibitor tocilizumab in Behçet’s disease with refractory arterial involvement indicated that tocilizumab is both safe and effective, additionally reducing the necessity for steroids and immunosuppressants.^[62]^

For patients considered high risk for major bleeding, embolization is preferred over open surgical intervention. In cases involving aortic or peripheral artery aneurysms, it is crucial to manage with cyclophosphamide and corticosteroids before any surgical repair. However, in symptomatic patients, surgical intervention or stenting should not be delayed.^[58]^

### 6.4. Neurological involvement

The 2014 expert consensus on Behçet’s disease recommends initiating treatment with corticosteroids, preferably through intravenous methylprednisolone, administered for 3 to 10 days, followed by an oral corticosteroid regimen lasting up to 6 months. If there is a significant parenchymal relapse, it is advised to consider disease-modifying therapy, taking into account factors such as disease severity, response to steroids, history of neurological relapses, and systemic features of Behçet’s disease. Azathioprine is established as the first-line disease-modifying therapy, with alternative agents including mycophenolate mofetil, methotrexate, and cyclophosphamide.^[48]^

Biologic agents, including TNF-alpha blockers (infliximab, adalimumab, etanercept) and interferon-alpha, should be considered when first-line treatments are ineffective or intolerable, particularly in cases characterized by relapses or aggressive disease progression. Cyclosporine, given its potential neurological complications, should be used with caution; it is contraindicated in patients with neuro-Behçet’s disease (NBD). In instances where neurological symptoms indicate central nervous system involvement, cyclosporine should be promptly discontinued.^[48]^

For patients with cerebral venous thrombosis associated with Behçet’s disease, corticosteroids are recommended for acute and sub-acute cases; however, evidence regarding the use of anticoagulants remains inconclusive. Anticoagulation should only be considered once systemic aneurysms have been ruled out. Poor prognostic indicators in NBD include involvement of the brainstem or myelopathy, frequent relapses, rapid disease progression, and cerebrospinal fluid pleocytosis, which necessitate the early initiation of disease-modifying therapy in these patients.^[48]^

A multicenter study involving 30 patients who met the international criteria for Behçet’s disease found that tocilizumab may be an effective alternative to anti-TNF agents in managing Behçet’s disease-related neurological symptoms and uveitis.^[63]^

### 6.5. Articular involvement

A clinical trial evaluated the effectiveness of colchicine in treating arthritis associated with Behçet’s disease by comparing it to a placebo in a group of 116 patients. During a 2-year follow-up period, those who received colchicine showed a significant decrease in the number of affected joints.^[64]^

For patients with refractory or persistent arthritis related to Behçet’s disease, azathioprine and/or TNF-alpha inhibitors are preferred over colchicine. A 2-year, randomized, placebo-controlled, double-blind trial conducted in Turkish men with Behçet’s disease (excluding those with eye involvement) demonstrated that azathioprine (2.5 mg/kg daily) was significantly more effective than placebo in preventing the onset of new ocular disease. Additionally, patients treated with azathioprine showed a reduction in the occurrence of oral ulcers, genital ulcers, and episodes of arthritis.^[65]^ Anti-TNF therapies, including infliximab and adalimumab, have also been effective in managing severe and resistant manifestations of Behçet’s disease, with similar efficacy observed among different TNF inhibitors.^[66]^

### 6.6. Gastrointestinal involvement

Gastrointestinal involvement in Behçet’s disease should be assessed through endoscopy and/or imaging studies. Before confirming the diagnosis, it is crucial to exclude conditions such as NSAID-induced ulcers, inflammatory bowel disease, and infections like tuberculosis.^[58]^

A retrospective study indicated that azathioprine is used as the initial treatment for moderate to severe cases, while 5-aminosalicylic acid (5-ASA) is used for milder cases. In patients with refractory disease, remission was achieved in 67% of cases through the administration of anti-TNF therapy and/or thalidomide. Azathioprine has shown effectiveness as a first-line treatment, providing high remission rates with minimal side effects. In resistant cases, thalidomide and/or TNF-alpha inhibitors may be preferred.^[67]^

Furthermore, a retrospective analysis of medical records from 8 tertiary hospitals in Korea revealed that infliximab was well-tolerated and effective in managing patients with moderate to severe intestinal Behçet’s disease.^[68]^

## 7. Therapy update

A phase 2 randomized controlled trial compared infliximab and cyclophosphamide for induction therapy in severe Behçet’s syndrome. By week 22, infliximab resulted in a higher complete response rate (81% vs 56%) and fewer adverse events (29.6% vs 64%) compared to cyclophosphamide. Serious adverse events were similar between groups. Infliximab was found to be more effective and safer for induction treatment.^[69]^

Another head-to-head randomized trial compared infliximab and interferon alfa-2a (IFN-α2a) in patients with refractory Behçet’s syndrome, as defined by the UK Centres of Excellence, which is characterized by failure to respond to steroid and/or immunomodulatory therapy, alongside significant or major organ-threatening disease. Both treatments demonstrated comparable clinical efficacy and safety at 12 and 24 weeks, with no significant difference in disease activity or symptom improvement noted. Steroid use decreased in both groups, with a slightly greater decrease in the IFN-α2a group. Infliximab showed a trend toward better tolerability and treatment persistence.^[70]^

Additionally, a head-to-head trial compared ciclosporin, interferon alfa-2a, and adalimumab, each combined with corticosteroids, for preventing uveitis relapse in patients with severe Behçet’s disease. Adalimumab was superior to ciclosporin, showing a significantly lower annualized uveitis relapse rate. Interferon alfa-2a was not found to be noninferior to adalimumab nor superior to ciclosporin. Serious adverse events occurred in all groups but were slightly more frequent with ciclosporin. These findings support the use of adalimumab as a more effective option for preventing uveitis relapse in patients naive to anti-TNF therapy.^[71]^

## 8. Gaps in knowledge, future perspectives, and directions

Behçet’s disease remains poorly understood, with key gaps in its pathogenesis, diagnosis, and treatment. The exact cause is unknown, though genetic and environmental factors are implicated. Clinical variability across regions hinders the standardization of diagnosis and management.

Current treatments control symptoms but lack curative potential, and data on newer biologics remain limited. The absence of specific biomarkers also delays diagnosis and complicates disease monitoring.

Future research should focus on identifying reliable biomarkers, clarifying immune mechanisms, and developing targeted, personalized therapies. Large, multinational studies are needed to address geographic variability and refine diagnostic and therapeutic strategies.

Supplemental digital content “RightsLink Printable License copy” is available for this article (https://links.lww.com/MD/Q874).

## Author contributions

**Data curation**: Mansour Alghamdi.

**Supervision**: Stephen Lindsey.

**Writing – original draft**: Mansour Alghamdi.

**Writing – review & editing**: Mansour Alghamdi.

## Supplementary Material
