# Supplementary material for: Behçet’s disease unraveled: Insights into clinical manifestations, diagnosis, and management
Source: Medicine (Baltimore). 2026 May 12;104(49):e44614. doi: 10.1097/MD.0000000000044614 (PMC12688949; doi:10.1097/MD.0000000000044614)
Supplement: Supplementary file 1 [file medi-104-e44614-s001.pdf]

MASSACHUSETTS MEDICAL SOCIETY LICENSE
TERMS AND CONDITIONS

Jul 23, 2025

This Agreement between Mansour Alghamdi ("You") and Massachusetts Medical Society ("Massachusetts Medical Society") consists of your license details and the terms and conditions provided by Massachusetts Medical Society and Copyright Clearance Center.

All payments must be made in full to CCC. For payment instructions, please see information listed at the bottom of this form.

License Number	6075080861718
License date	Jul 23, 2025
Licensed Content Publisher	Massachusetts Medical Society
Licensed Content Publication	The New England Journal of Medicine
Licensed Content Title	Behçet’s Syndrome
Licensed Content Author	David Saadoun, Bahram Bodaghi, Patrice Cacoub
Licensed Content Date	Feb 15, 2024
Licensed Content Volume	390
Licensed Content Issue	7
Type of Use	Journal/Magazine
Requestor type	academic/educational
Portion	figure/table

Number of figures/tables	1
Would you like a high resolution image with your order?	No
Format	electronic
Will you be translating?	no
Circulation	1000000
Distributing to	Worldwide
Title of new article	Behçet's Disease Unraveled: Insights into Clinical Manifestations, Diagnosis, Treatment, and Management
Lead author	Mansour Alghamdi
Title of targeted journal	Medicine
Publisher	Wolters Kluwer Health, LWW
Expected publication date	Sep 2025
Portions	Figure 3. Clinical Manifestations of Behçet's Syndrome.
The Requesting Person / Organization to Appear on the License	Mansour Alghamdi
Requestor Location	Dr. Mansour Alghamdi<br>5036 HASTINGS ST<br><br>METAIRIE, LA 70006<br>United States

Billing Type	Credit Card
Credit card info	American Express ending in 0015
Credit card expiration	07/2028
Total	64.96 USD

Terms and Conditions

Introduction

The publisher for this copyrighted content is Massachusetts Medical Society ("MMS"). By clicking "accept" in connection with completing this licensing transaction, you agree that the following terms and conditions apply (along with the Billing and Payment terms and conditions established by Copyright Clearance Center, Inc. ("CCC"), at the time that you opened your RightsLink account and that are available at any time at http://myaccount.copyright.com).

Scope of License

1. MMS hereby grants to you a non-exclusive license to reproduce the aforementioned content subject to the terms and conditions indicated within the RightsLink transaction. Licenses are for one-time use only. Web posting is limited to the time period selected within your transaction - beginning on the date of this license.

2. This grant of permission is for the content to be used without alteration, as originally published by MMS. However, minor formatting and stylistic changes are permitted, provided that any explanatory material or figure legend that you use accurately reflects the content as originally published by MMS. MMS does not approve adaptations or modifications.

3. MMS's copyrighted content may not be used in any manner that implies endorsement, sponsorship, or promotion of any entity, product or service by the MMS or its publications. MMS cannot and does not authorize the use of any author's name on promotional materials; such approval must be obtained directly from the author.

4. This permission is granted for English language rights only unless your license specifically grants translation rights. If translation rights are granted, the content must be reproduced word for word with the integrity and original meaning of the content preserved.

5. If your license specifically grants "expanded rights," permission is granted to reproduce the aforementioned content in a primary work and related materials created to supplement or add value to it, such as: ancillaries, instructor guides, testing materials, and student resource materials.

6. MMS reserves all rights not specifically granted in the combination of (i) the license details provided by you and accepted in the course of this licensing transaction, (ii) these

terms and conditions and (iii) CCC's Billing and Payment terms and conditions.

Acknowledgement

7. This permission requires suitable acknowledgement of the source, either as a footnote, in a reference list, or adjacent to where the MMS content appears. The credit line should read as follows:
"From [Publication Title, Author(s), Title of Article, Volume No., Page No. Copyright © (notice year) Massachusetts Medical Society. Reprinted with permission from Massachusetts Medical Society."

General Terms

8. Warranties. MMS makes no representations or warranties with respect to the licensed content.

9. Indemnity. You hereby indemnify and agree to hold harmless the MMS and CCC, and their respective officers, directors, employees, members and agents, from and against any and all claims arising out of your use of the licensed content other than as specifically authorized by this license.

10. No Transfer of License. This license is personal to you and may not be sublicensed, assigned, or transferred by you to any other person or entity without permission from MMS.

11. No Amendment Except in Writing. This license may not be amended except in a writing signed by both parties.

12. Objection to Contrary Terms. MMS hereby objects to any terms contained in any purchase order, acknowledgment, check endorsement or other writing prepared by you, which terms are inconsistent with these terms and conditions or CCC's Billing and Payment terms and conditions. These terms and conditions, together with CCC's Billing and Payment terms and conditions (which are incorporated herein), comprise the entire agreement between you and publisher (and CCC) concerning this licensing transaction. In the event of any conflict between your obligations established by these terms and conditions and those established by CCC's Billing and Payment terms and conditions, these terms and conditions shall control.

13. License Contingent on Payment. While you may exercise the rights licensed immediately upon issuance of the license at the end of the licensing process for the transaction, provided that you have disclosed complete and accurate details of your proposed use, no license is finally effective unless and until full payment is received from you (either by MMS or by CCC) as provided in CCC's Billing and Payment terms and conditions. If full payment is not received on a timely basis, then any license preliminarily granted shall be deemed automatically revoked and shall be void as if never granted. Further, in the event that you breach any of these terms and conditions or any of CCC's Billing and Payment terms and conditions, the license is automatically revoked and shall be void as if never granted. Use of content as described in a revoked license, as well as any use of the content beyond the scope of an unrevoked license, may constitute copyright infringement and MMS reserves the right to take any and all action to protect its copyright in the content.

14. This license transaction shall be governed by and construed in accordance with the laws of the Commonwealth of Massachusetts. You hereby agree to submit to the

jurisdiction of the federal and state courts located in Boston, MA for purposes of resolving any dispute that may arise in connection with this licensing transaction.

Restrictions

15. This permission applies only to content of the MMS, and not to copyrighted content from any other source. If content appears in our work with credit to another source, you must also obtain permission from that source.

16. This permission does not apply to and is not valid for photographs depicting identifiable individuals.

Other Terms and Conditions:

5_16_12_v1.0

Questions? customercare@copyright.com.
